# Fine-scale selection by ovipositing females increases egg survival

**DOI:** 10.1002/ece3.389

**Published:** 2012-10-01

**Authors:** Brian G Gall, Edmund D Brodie, Edmund D Brodie

**Affiliations:** 1 Department of Biology, Hanover CollegeP.O. Box 108, Hanover, Indiana, 47243; 2 Mountain Lake Biological Station and Department of Biology, University of VirginiaP.O. Box 400328, Charlottesville, Virginia, 22904; 3 Department of Biology, Utah State University5305 Old Main Hill, Logan, Utah, 84322

**Keywords:** Choice, habitat selection, oviposition, *Taricha granulosa*, tetrodotoxin

## Abstract

One of the most important defenses for the eggs of ovipositing female organisms is to avoid being laid in the same habitat as their predators. However, for most organisms, completely avoiding an offspring's predators is not possible. One mechanism that has been largely overlooked is for females to partition an oviposition site into microhabitats that differ in quality for offspring survival. We conducted a series of experiments to examine whether female newts avoid microhabitats utilized by their offspring's primary predator, caddisfly larvae. Female newts avoided laying eggs near predatory caddisflies and shifted egg laying upward in the water column when provided with a vertical dimension. Caddisflies were attracted to chemical stimuli from female newts and their eggs, yet primarily used benthic areas in experimental chambers. Finally, results from a field experiment indicate that the behavioral strategy employed by female newts increases offspring survival. This subset of non-genetic maternal effects, micro-oviposition avoidance, is likely an important yet underexplored mechanism by which females increase offspring survival.

## Introduction

Where a female deposits her eggs can have dramatic consequences on her immediate reproductive success and total lifetime fitness. Abiotic characteristics such as temperature, humidity, water level, oxygen concentration, and nutrient composition often differ at relatively small spatial scales, thus creating oviposition sites that differ in quality (e.g. Potts and Willmer 1997; Rudolf and Rödel 2005). In addition, biotic interactions such as competition and predation can influence hatchling phenotype, growth, performance, and survival, which may subsequently have positive or negative impacts on female fitness (e.g. Morin 1986; Relyea 2004, 2007). It has become increasingly clear that non-genetic maternal effects, especially the quality of the oviposition site, can be as important as the genetic quality of the male or the allocation of nutrient resources in the egg prior to deposition (Kirkpatrick and Lande 1989; Resetarits 1996).

The importance of active choice by oviparous female organisms during egg deposition has been well established in numerous species. For example, many female amphibians sample oviposition sites and choose to deposit eggs in pools that lack egg and larval predators (Resetarits 1996; Resetarits and Wilbur 1989; von May et al. 2009). Female butterflies often sample multiple plant species during oviposition (Wiklund 1981; Singer 1983; Thompson 1988), choosing to lay eggs on the species that maximize larval growth (Rausher 1982; Singer et al. 1988; Thompson and Pellmyr 1991). Some female insects prefer to oviposit on plants with the greatest concentration of secondary metabolites, which are then sequestered by the developing larvae and function in defense against potential predators (Pereyra and Bowers 1988; Nishida 2002). Many female organisms have very precise discriminatory abilities when choosing oviposition sites. For example, the predatory midge *Aphidoletes aphidimyza* can detect a single plant that contains prey for its larvae out of 75 non-infested plants (El-Titi 1972 as in Lucas and Brodeur 1999). Female *Hyla femoralis* can detect chemical stimuli from a single 2-g predatory fish in a 400-L pool, subsequently causing them to avoid this pool and deposit eggs in predator-free sites (Rieger et al. 2004).

A female's opportunity to choose where to lay an egg does not stop once a general oviposition site has been selected. Many microhabitats exist within an oviposition location, and selection is likely to yield mechanisms whereby each egg, or group of eggs, is placed in a specific microhabitat that further enhances survival. Furthermore, ecological conditions experienced during the period of oviposition can change rapidly and females likely adjust their behavior according to these changing conditions. Despite a large volume of research on the ability of oviparous organisms to discriminate between discrete oviposition sites, and apart from a few studies on insects (e.g. Williams 1981; Lucas and Brodeur 1999; Hirayama and Kasuya 2009), very little empirical work has tested whether maternal behavior can influence offspring fitness through the selection of a microenvironment *within* an oviposition site. This underexplored mechanism of indirect maternal effects, micro-oviposition behavior, has the potential to affect the evolution of other phenotypic traits such as oviposition site selection (in the broad sense), host-plant shifts, and sequestration or synthesis of defensive compounds.

To examine for the presence of micro-oviposition behavior and determine whether this mechanism can influence offspring fitness, we conducted a comprehensive series of experiments examining the micro-oviposition behavior of a salamander (*Taricha granulosa*), the behavior and space use of a voracious egg predator (caddisfly larvae: *Limnephilus flavastellus*), and the fitness benefits of micro-oviposition avoidance behavior in a natural pond. After mating, the newt, *T. granulosa*, deposits eggs over a period of weeks to months beginning in early spring (Nussbaum et al. 1983). Each egg is deposited singly and attached to aquatic vegetation. In addition, newts, and their eggs, possess the powerful neurotoxin tetrodotoxin (TTX) (Mosher et al. 1964; Hanifin et al. 2003; Gall et al. 2012), which successfully repels almost all potential predators (Brodie 1968). A major source of mortality for newt eggs, despite their toxicity, is predatory caddisfly larvae that appear to be resistant to the negative effects of TTX ingestion (Gall et al. 2011). This system provides an ideal opportunity to test for differences in microhabitat selection by an ovipositing organism as influenced by predation, because the interface of selection is likely to be the result of direct interactions between a single predator and its prey. Moreover, maternal behavior is unlikely to be influenced by other factors, such as larval food requirements or male behavior, which are common in many phytophagous insects and anurans.

## Methods

### Animal collection

Male and female newts (*T. granulosa*) were collected in March 2009 and 2010 from Soap Creek ponds in Benton County, Oregon. Soap Creek consists of eight manmade ponds arranged in two rows of four and can be considered a single population (Gall et al. 2011; Hopkins et al. 2012). Females were collected from three adjoining ponds. Newts were transported to Utah State University and housed individually in 5.7-L plastic containers with 3 L of filtered tap water. They were maintained at 6°C to prevent spontaneous egg deposition and fed blackworms (*Lumbriculus variegatus*) weekly.

Caddisfly larvae (*L. flavastellus*, henceforth: caddisflies) were collected from the same ponds as *Taricha*. Caddisflies were housed in 37-L aerated aquaria with 20 L filtered tap water at 6°C. Caddisflies were fed maple-leaf detritus (see Gall et al. 2011 for a description of detritus preparation). Mayfly larvae (Baetidae; henceforth: mayflies) were used as a non-predatory control. Mayflies co-occur with *Taricha* at Soap Creek ponds, but at low densities. Mayflies were collected near Paradise, Utah, and housed in a 37-L aerated aquarium with a small amount of detritus. Except when serving as the source of the chemical stimuli for a treatment (see below), no caddisfly, mayfly, newt, or newt egg was reused for any experiment. All experiments were carried out within 1 year and never continued into the next season. Furthermore, the animals used in these experiments were used in the same year in which they were collected.

### Do caddisflies respond behaviorally to newts?

We examined the behavior of caddisflies to stimuli that they may be exposed to before or during a predatory encounter with newt eggs. Using two types of choice experiments, caddisflies were exposed to stimuli from (1) a blank control, detritus (food), male newts, and gravid and “spent” female newts, as well as (2) newt eggs and agar containing TTX.

#### Flow-through trials

The first set of trials exposed caddisflies to chemical cues in a flow-through test chamber consisting of a series of vertically positioned containers. The uppermost tub of the flow-through apparatus consisted of a 40-L reservoir that drained via two plastic tubes (3 mm ID) into two separate 5.7 L stimulus containers. These stimulus containers then drained into two sides of a testing container separated by a plastic partition that prevented the stimuli from mixing until they passed through mesh and into the experimental chamber (4 × 16.6 × 5 cm). Water flowed between containers at 0.4 L/min. As effluent passed through the mesh and out drains at the back of the experimental chamber, a chemical gradient was produced preventing the stimuli from mixing while permitting caddisflies to move freely between the two chemical zones. Preliminary trials using dye indicated that less than 30 sec was required for the stimuli to disperse and the gradient to become established. The bottom of the test chamber was lined with a thin layer of course sand to provide a substrate for caddisflies to grasp. All trials were conducted inside an environmental chamber at 12°C.

A single *L. flavastellus* was exposed to chemical stimuli from one of six sources: (a) control (double blank), (b) detritus (food), (c) recently deposited newt eggs, (d) male newts in reproductive condition, (e) gravid female newts, or (f) “spent” female newts that had completed egg deposition (*N* = 10 trials per treatment). The detritus treatment was prepared with 30 g of conditioned detritus, which was gently rinsed with filtered water. Eggs (*N* = 1272) used in the newt-egg treatment were deposited in polyester filter fiber by five female newts not used in this study. The eggs and fiber were rinsed thoroughly to remove any female newt cues. The male newts were in reproductive condition. The female newts were gravid and had not yet begun depositing the bulk of their eggs. They laid several eggs prior to testing and continued laying eggs after testing; however, no eggs were deposited during testing. To test spent female newts, these same two females were each injected with 2μL/g LHRH (de-Gly10, [d-His(Bzl)6]-Luteinizing Hormone Releasing Hormone Ethylamide; Sigma #12761, Sigma-Aldrich, St. Louis, Missouri). The spent female treatment was conducted 2 weeks following the conclusion of egg deposition by these females.

All stimulus animals and caddisflies were transferred to the environmental chamber 24 h prior to testing to acclimate to the higher temperature. Caddisflies were not deprived of food prior to testing. Inside the environmental chamber, a large reservoir was maintained with filtered tap water. Thirty minutes prior to the beginning of each trial, the stimulus containers were filled with 2 L of water and a treatment was randomly placed into one of the stimulus containers, while the other container was left empty as a blank control. The testing chamber was then filled with 400 mL of water. After a 30-min acclimation period, one caddisfly larva was placed in the center of the test container. The flow was immediately initiated beginning with the uppermost tub, and the caddisfly larva was allowed to acclimate for 3 min. After the acclimation period, we recorded the position (control or chemical stimulus) of the caddisfly in the experimental chamber every 30 sec and the number of times the caddisfly crossed the center line. Observations were made for 20 min. After each trial, the caddisfly larvae were weighed to the nearest 0.01 g, and the stimulus containers, plastic tubing, and experimental chamber were thoroughly rinsed with warm filtered water.

#### Static water test chamber

A second set of trials was conducted in an arena with the stimulus source placed directly in the tank. These trials were used to verify the trend toward egg attraction observed in the first choice trials (see Results) and determine whether TTX was used as a cue to locate the eggs. The test chamber consisted of a 9 × 3 × 2.3 cm plastic container with a line drawn across the middle to separate it into two halves. The caps from two 1.5 mL screw-cap tubes were inverted and glued 1.5 cm from each end of the container. Sixty small holes were punched in each tube to permit the passage of chemical stimuli, but prevent caddisflies from accessing the eggs or agar inside.

Individual caddisflies were exposed to 30 newt eggs and a blank control (*N* = 26), or agar containing 46 μg TTX (equivalent to the average amount of TTX present in 30 newt eggs) and control agar (*N* = 20). The test chamber was filled with 50 mL filtered tap water. A centrifuge tube was filled with either 30 newt eggs or agar containing TTX and screwed to a randomly chosen cap (see below for agar preparation). A second centrifuge tube was screwed to the other cap, but left empty (if paired with newt eggs) or filled with control agar (if paired with agar containing TTX) to serve as a control. After 10 min, a caddisfly was placed inside an acclimation cylinder (2.7 cm diameter) in the center of the test chamber for 3 min. The cylinder was then removed and trial initiated. We recorded the position (control or chemical stimulus) of the caddisfly in the experimental chamber every 30 sec and the number of times the caddisfly crossed the center line. Observations were made for 20 min. The test chamber was rinsed with warm water after each trial. The number of observations spent on each side of the test tank (control or treatment) was tabulated for each caddisfly and divided by the total number of possible observations. The proportion of observations on the stimulus side of the test container was compared with a random distribution of 0.50 using a one-sample *t*-test. The number of lines crossed in each treatment was compared using an analysis of variance (ANOVA) followed by Holm–Sidak multiple comparisons.

#### Preparation of agar containing TTX

Because of the presence of extreme TTX levels in our experimental eggs, we added TTX to agar to determine if caddisflies are specifically attracted to this toxin (*N* = 20). The average amount of TTX/egg, excluding the jelly coat, from the Soap Creek newt population is 1.528 μg. The volume of an egg (excluding the jelly coat) from this population ranges from 4.92 to 8.67 μL (C. Hanifin, pers. comm.); we used a volume of 7 μL/egg to calculate the average volume and amount of TTX in 30 eggs.

We made both control and TTX containing agar using Ionagar No. 2 (Consolidated Laboratories, Inc., Chicago Heights, IL). Control agar was made by mixing 1.5 g agar with 100 mL boiling distilled water. After the solution had partially cooled, it was poured into a Petri dish. The agar was then allowed to cool and solidify, at which time it was placed in a refrigerator. Because an extremely large quantity of TTX is present in the eggs of newts from this population, we made substantially less TTX containing agar compared with the blank control. We mixed 2 mg TTX with 1 mL distilled water, then boiled 9 mL distilled water and added 0.15 g agar. After the boiled water-agar solution had cooled, but not solidified, we added the TTX solution, mixed the solution thoroughly, and poured it into a Petri dish. It was then allowed to solidify and was refrigerated. A punch was used to remove a section of agar that was equal to the volume of 30 newt eggs. Separate punches were used for the two agars, which were refrigerated between punch removal.

### Do newts possess strategies that limit predation on their eggs?

To determine if newts possess behavioral strategies that limit predation on their eggs, we recorded the oviposition behavior of female newts in response to caddisflies, examined the microhabitat use of larval caddisflies, and conducted a field study to determine whether the behavioral strategy employed by female newts increased egg survival.

#### Oviposition choice

**Figure 1:**
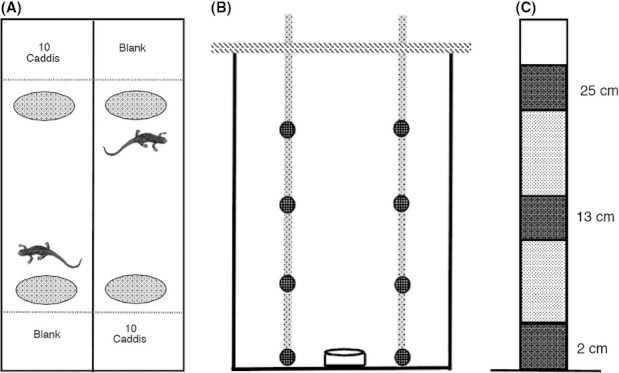
(A) Experimental chamber used to test ovipositing female newts' responses to the presence of caddisflies and mayflies. Screen (dashed line) prevented the invertebrates from interacting with the female or consuming eggs. Clumps of polyester fiber (ovals) were provided for oviposition sites. (B) Vertical chamber used to test the responses of ovipositing female newts to the presence of caddisflies, mayflies, or a blank control. Oviposition sites consisted of polyester clumps (dark circles) that were attached to willow (*Salix amygdaloides*) branches at 0, 9.5, 19.0, and 28.5 cm above the floor of the chamber. Invertebrates were maintained in two clear cylinders with screen tops on the bottom of the bucket. (C) Experimental stake used in a field experiment testing newt-egg survival at three different heights above the pond substrate. Eggs were attached to one of three turf squares (dark stippling) and separated by rectangular pieces of turf (light stippling). The stake was pushed into the pond 20 degrees from vertical, such that the bottom of the lowest square rested on top of the substrate.

A choice test was used to determine the propensity of female newts to avoid ovipositing near predatory caddisflies (*N* = 8 trials) or non-predatory mayflies (*N* = 6 trials). Female newts were tested in one half of a 74-L aquarium divided lengthwise by a piece of opaque plexi-glass that prevented water exchange between the two halves of the test tank (Fig. 1A). A piece of screen (10 × 15 cm, 1.5 mm mesh) was glued 7 cm from each end. Polyester fiber (5 × 10 cm) was anchored to a suction cup at each end of the middle compartment to serve as egg deposition sites. Females were able to move freely between fiber blocks and choose between oviposition sites.

Three aquaria and six experimental chambers were run simultaneously. Each test tank was filled with 6 L of filtered tap water. Ten caddisflies or mayflies were randomly assigned to one of the small compartments with the second compartment remaining empty thereby creating a treatment side and a control side (Fig 1A). The second test tank (within the same aquarium) was assigned the opposite treatment structure as the first tank.

A female newt was injected 2 μL/g LHRH and placed in the test tank. Females were monitored every 2 h from 0800 to 2000 h to determine the beginning of egg deposition. Newts were removed from the test tank 24 h after the beginning of egg deposition, and the number of eggs on each piece of filter fiber was counted. We made an a priori decision to remove females from the analysis if fewer than 50 eggs were deposited in the 24 h test period because these females may not have completely entered oviposition and may require an additional injection of LHRH. The experimental chambers were emptied and rinsed with hot water after each trial. The number of eggs deposited on the control and treatment sides of the test tank was compared with a paired t-test. Assumptions for parametric statistics were met by these data.

#### Oviposition behavior in vertical chamber

We tested the responses of ovipositing females in a vertical test chamber to chemical stimuli from predatory caddisflies (*N* = 10), non-predatory mayflies (*N* = 10), and a blank control (*N* = 10). Each test chamber consisted of a 19-L bucket. Oviposition sites were provided at 0, 9.5, 19.0, and 28.5 cm along a vertical axis starting at the bottom of the bucket (Fig. 1B). Each oviposition site consisted of a 1 g piece of polyester fiber glued to a willow (*Salix amygdaloides*) branch at the appropriate height. Two branches with oviposition sites were present in each bucket (Fig 1B) and each bucket was filled to a height of 32 cm with filtered tap water.

Caddisflies or mayflies were held in two containers at the bottom of the test chamber (Fig. 1B). These containers consisted of a 3-cm long piece of clear plastic tubing (8.9 cm diameter) with a cap on the bottom. Each container was filled with eight conditioned maple leaves (*Acer grandidentatum*) and five of the appropriate invertebrates or no invertebrate (control). A piece of fiberglass window screen was fixed to the top of the container with an elastic band. Two of these containers of the appropriate treatment were placed in the bottom of each bucket. A gravid female newt was randomly assigned to one of the three treatments, injected 2μL/g LHRH, and placed in the bottom of the bucket. Each chamber was assembled immediately prior to the start of each trial to prevent individual females from experiencing different gradients of chemical cues at the beginning. Trials were conducted inside an environmental chamber at 11°C with 12L:12D. Females were monitored daily for the beginning of egg deposition. After approximately 50 eggs had been deposited, the female was removed and the total number of eggs on each fiber block was counted. When trials were terminated (24–48 h after the start of oviposition), all females had deposited more than 50 eggs.

To compare egg deposition between treatments, we analyzed the proportion of eggs deposited at each height relative to the total number of eggs deposited. Data were analyzed with a generalized linear mixed model using number of eggs as the response and (log-transformed) total number of eggs deposited as an offset, with a negative binomial distribution and a log link. The design structure partitioned variance between and within females in a split-plot design, with female as the whole plot unit, a repeated measure (within a female) as the subplot unit, and treatment and height as the two fixed-effects whole plot and subplot factors, respectively. Analyses were obtained using the GLIMMIX procedure in SAS 9.2 (SAS Institute Inc., Cary, NC). We also compared the mean total number of eggs deposited in each treatment with an ANOVA.

#### Caddisfly distribution in relation to oviposition behavior

We examined the vertical space use of *L. flavastellus* in aquatic vegetation. Three different species of plants were used (*Vallisneria americana, Egeria densa, Bacopa monnieri*); however, plant type had no effect on the results and will not be discussed further. The test chamber consisted of a 3.8-L glass jar with 4.5 cm of course sand and 3.5 L of filtered tap water. Four lines were drawn around the jar every 4.5 cm from the top of the sand, resulting in four zones of increasing height, as well as the ground zone (located on the substrate). Three plants of a single species were placed in the sand in a triangular array. Four conditioned maple leaves and five caddisflies were placed on the substrate. After a 20-min acclimation period, we recorded the position of each caddisfly within each zone [ground (0 cm), 0–4.5 cm, 4.5–9 cm, 9–13.5 cm, 13.5–18 cm] every 20 min for 5 h. Two replicates were conducted per plant species resulting in six experimental chambers. The total number of caddisfly observations at each height was summed for each experimental container. These data were analyzed by a two-way ANOVA with plant type and height as the two fixed factors. Data were square-root transformed to meet assumptions of normality.

We also measured the vertical space use of individual caddisflies to determine what role size (mass, case length, and case width) had on the height obtained in aquatic vegetation (*Egeria densa*) (*N* = 26). The experimental chamber and test procedure were the same as previously described, except that a single caddisfly was placed in each jar. At the conclusion of testing, we recorded the length and diameter of the case as well as the mass of the caddisfly (without case). We calculated the mean height obtained by the caddisfly during each trial by assigning each zone a value based on the distance from the middle of that zone to the substrate and averaging all the observations from the 5-h trial. We compared case length, case diameter, and caddisfly mass with the mean height obtained during the trials with linear regression.

#### Egg survival – field experiment

We tested the survival of newt eggs positioned at one of three heights (2, 13, or 25 cm) above the substrate in a natural pond. Gravid female newts (*N* = 10) were collected from Soap Creek ponds and transported to Corvallis, Oregon. Each female was injected 20 μL LHRH. A female was placed in a 15-L tub with approximately 5 L of pond water. Small squares (3.8 × 3.8 cm) of artificial turf were glued to ceramic tiles and placed in the bottom of the tub for females to oviposit on. After females had deposited five eggs on a small square, the square was removed and placed in a separate tub for transportation to the field site. At 0900 h, all squares (collected either at 2000 h the previous day or 0700 h that morning) were transported to the pond. The tub containing the experimental squares was placed in the pond to acclimate the eggs to the pond temperature.

One small square of turf containing five eggs was randomly assigned to the bottom, middle, or top of a 59-cm-long wooden stake (Fig 1C). Two small squares (without eggs) were stapled to the remaining empty positions, and two rectangular pieces of turf (3.8 × 17.5 cm) were stapled in the gaps, thus creating a continuous piece of turf with five eggs at the appropriate height (Fig 1C). An imaginary grid (seven rows and three columns; each square was approximately 3 × 3 m) was created across the pond and a stake was randomly assigned to one of the 21 positions. Stakes were pushed into the substrate (approximately 20 degrees from vertical) until the bottom of the lowest square rested on the substrate. Trials were initiated in three separate ponds (*N* = 5 or 6 stakes/treatment/pond). After 25 h, the stakes were removed and the number of surviving eggs was recorded.

We compared the number of surviving eggs on each stake among the three heights using a general linear model followed by REGWQ multiple comparisons in SAS v 9.1. Stake was treated as the experimental unit, with the number of surviving eggs counted for each stake. Height was treated as a fixed-effect factor, while pond was incorporated into the model as a random factor. These data were squared to meet assumptions of normality.

## Results

### Do caddisflies respond behaviorally to newts?

**Figure 2:**
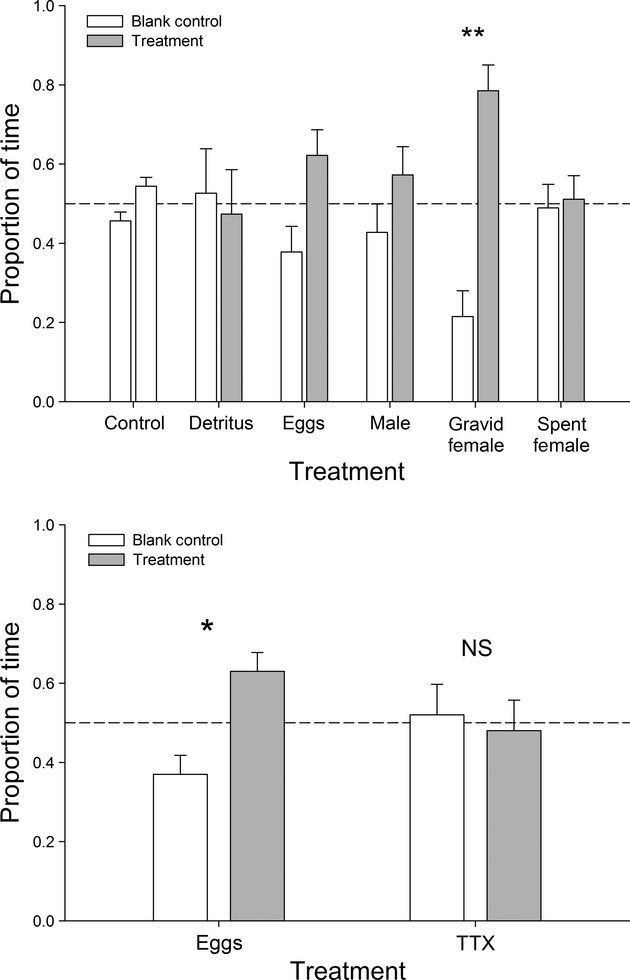
Do caddisflies respond behaviorally to newts? (Top) Proportion (Mean ± SE) of time caddisflies spent on the control and treatment side of a flow-through test chamber during choice trials. Dashed line indicates a random distribution. **P = 0.002, all other *P* > 0.09. (Bottom) Mean (±SE) proportion of time caddisflies spent on the control and treatment side of a stagnant-water test chamber during choice trials. Dashed line indicates a random distribution. *P < 0.05, NS = non-significant.

Caddisflies exposed to cues from gravid female newts and a blank control spent significantly more time on the side of the test container associated with the gravid female newts (*t* = 4.385, df = 9, *P* = 0.002; Fig. 2). However, several weeks after these females had deposited all of their eggs, caddisflies spent a similar portion of time on both sides of the test tank (*t* = 0.187, df = 8, *P* = 0.857; Fig. 2). When exposed to control, detritus, recently deposited eggs, and male newts, caddisflies exhibited a random distribution within the test tank (all *P* > 0.09; Fig. 2). There was no significant difference (and no apparent trend) between treatments in activity levels as measured by the number of lines crossed (H = 2.3, df = 5, *P* = 0.06).

Caddisflies exposed to newt eggs and a blank control in a static water container spent significantly more time in the portion of the test chamber containing newt eggs (*t* = 2.73, df = 25, *P* = 0.011; Fig. 2). However, caddisflies were not specifically attracted to agar containing 46 μg of TTX (*t* = −0.261, df = 19, *P* = 0.80; Fig. 2). There was no significant difference in the number of lines crossed between caddisflies exposed to eggs or agar with TTX (*t* = 0.53, df = 44, *P* = 0.60).

### Do newts possess strategies that limit predation on their eggs?

#### Oviposition choice

**Figure 3:**
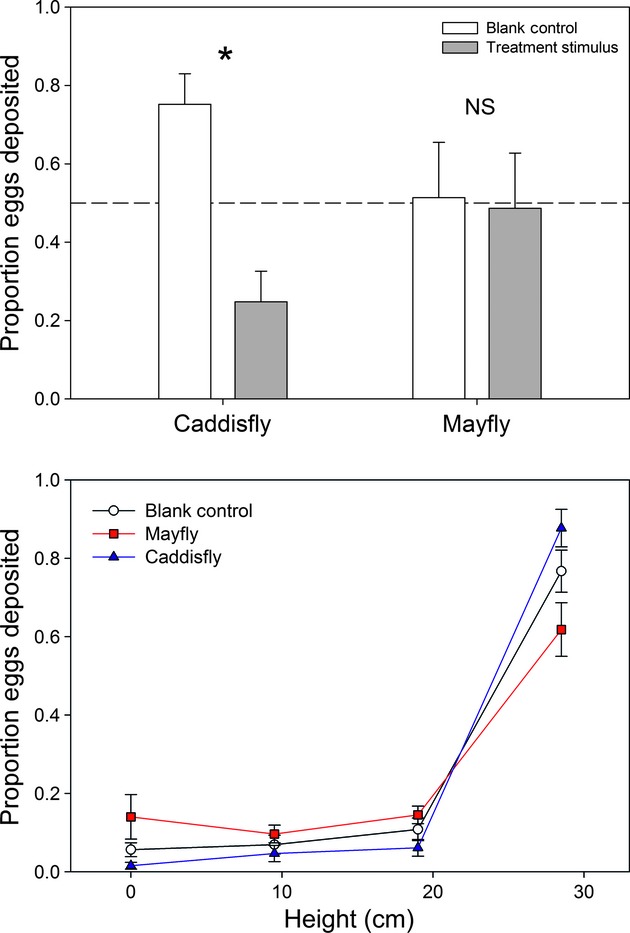
Do newts possess strategies that limit predation on their eggs? (Top) Mean (±SE) percentage of eggs deposited by female newts on oviposition sites either next to (gray bar) or away from (white bar) predatory caddisflies or non-predatory mayflies. *P = 0.014; NS, *P* = 0.93. (Bottom) Mean (±SE) proportion of eggs deposited at four different heights (cm) by female newts exposed to predatory caddisflies (triangle), non-predatory mayflies (square), or a blank control (circle). Newts decrease the number of eggs deposited near the bottom and deposit more eggs near the top of the water column when an egg predator (caddisfly larvae) is placed near the bottom of the test chamber.

Ovipositing female newts responded strongly to caddisflies, depositing just 25% of their eggs near this predator and 75% on the control side of the test chamber (*t* = 3.233, df = 7, *P* = 0.014; Fig. 3). However, egg deposition between the two sides of the test tank in response to non-predatory mayflies did not differ from random (*t* = 0.10, df = 5, *P* = 0.93; Fig. 3).

#### Oviposition behavior in vertical chamber

Significant main effects were detected for both treatment (*F*_[2,28]_ = 7.51, *P* = 0.0024) and height (*F*_[3,80]_ = 65.71, *P* < 0.0001). A significant interaction effect between treatment and height was also identified (*F*_[6,80]_ = 3.79, *P* = 0.0023, Fig 3). When exposed to caddisflies, ovipositing females shifted deposition upward relative to females exposed to mayfly and control treatments (Fig. 3). Females exposed to caddisflies oviposited just 1.5% of all eggs on the bottom fiber block compared with 5.6% and 14.0% in the control and mayfly treatments, respectively. This shift away from the bottom resulted in 87.7% of all eggs being deposited at the top when exposed to caddisflies compared with 61% in the non-predator mayfly treatment and 76% in the control treatment. There was no difference between treatments in the mean total number of eggs deposited (Caddisfly: 81.2 ± 7.5; Mayfly = 77.5 ± 3.1; Control: 76.8 ± 3.7; *F* = 0.21, *P* = 0.81).

#### Caddisfly distribution in relation to oviposition behavior

**Figure 4:**
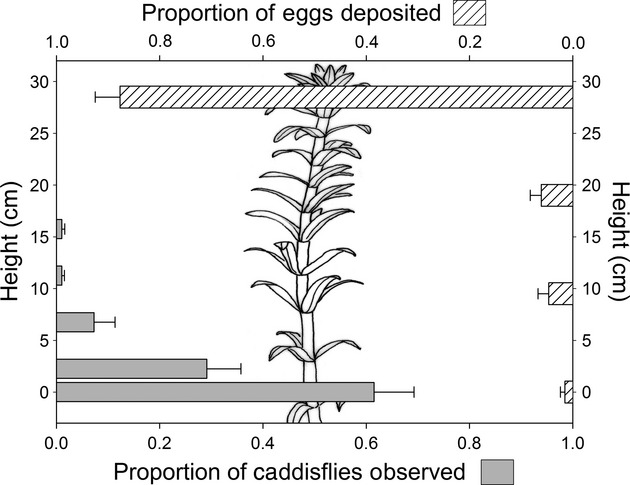
The number of caddisflies observed at five heights in aquatic vegetation over a 5-h period compared with the proportion of eggs deposited at four different heights by female newts exposed to caddisflies during vertical oviposition trials. All trials were conducted in the lab. *Elodea* line drawing provided by the University of Florida, Center for Aquatic and Invasive Plants.

There was a significant main effect of height on the distribution of caddisflies throughout the plants (*F*_[4,29]_ = 54.93, *P* < 0.001, Fig. 4). Caddisflies primarily utilized the substrate and lowest sections of vegetation and few were observed in the upper sections of vegetation; they generally did not utilize areas where newt eggs were likely to be deposited (Fig. 4).

**Figure 5:**
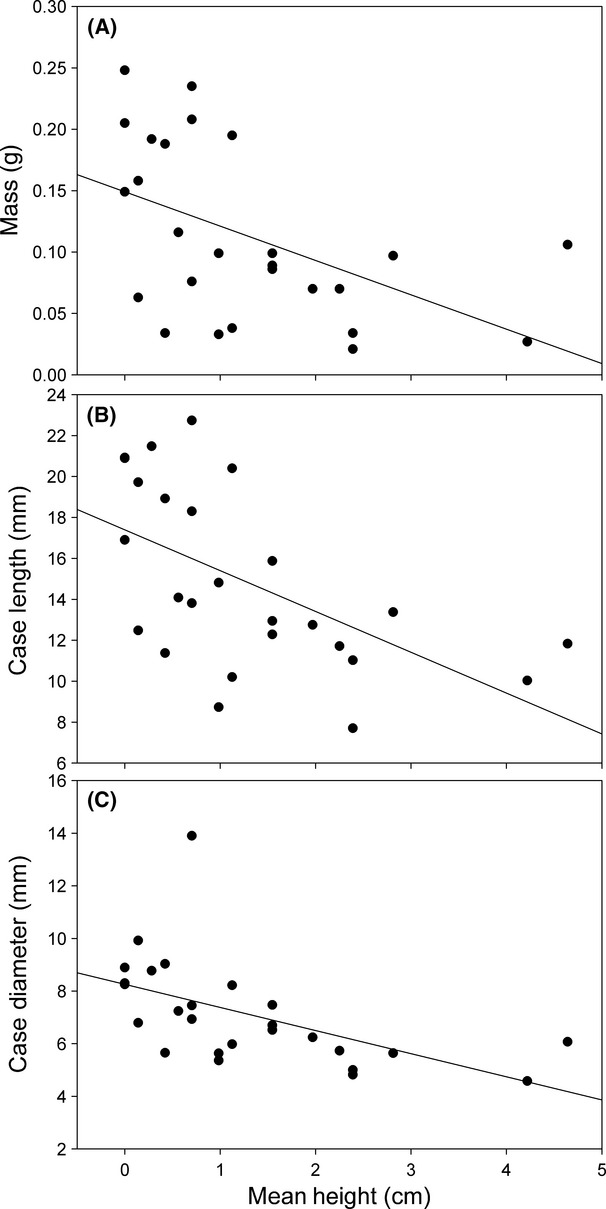
The mean height obtained in aquatic vegetation by larval caddisflies (*Limnephilus flavastellus*) in relation to (A) Larval mass (*F*_[1,25]_ = 6.1, *R*^2^ = 0.20, *P* = 0.02), (B) case length (*F*_[1,25]_ = 9.1, *R*^2^ = 0.27, *P* = 0.006), and (C) case diameter (*F*_[1,24]_ = 7.0, *R*^2^ = 0.22, *P* = 0.01).

Smaller caddisflies climbed higher in aquatic vegetation than large caddisflies, which remained on or close to the substrate (Fig 5). There was a significant negative relationship between caddisfly mass (*F*_[1,25]_ = 6.1, *R*^2^ = 0.20, *P* = 0.02, Fig 5A), case length (*F*_[1,25]_ = 9.1, *R*^2^ = 0.27, *P* = 0.006, Fig 5B), and case diameter (*F*_[1,25]_ = 7.0, *R*^2^ = 0.22, *P* = 0.01, Fig 5C) and the height obtained in vegetation.

#### Egg survival – field experiment

**Figure 6:**
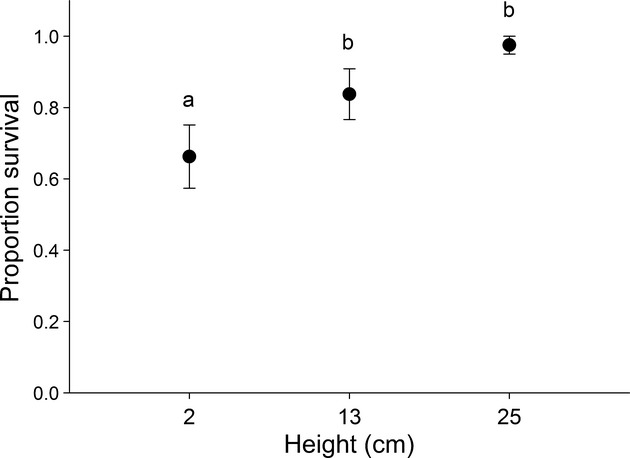
Mean (±SE) proportion of eggs that survived over a 25-h period when placed at one of three different heights (cm) above the substrate in a natural pond. Different letters indicate significant differences between treatments (*P* < 0.05).

Caddisflies were observed on the experimental stakes, and evidence of predation by caddisflies (torn egg jelly and consumed yolk) was identified on many stakes. The height of newt eggs in the pond had a significant effect on their survival (*F*_[4,43]_ = 7.51, *P* = 0.002, Fig 6), with eggs placed near the substrate suffering the greatest predation, and survival increasing with increasing height (Fig 6). Block (pond) had no effect on egg survival (*F*_[4,43]_ = 1.44, *P* = 0.25).

## Discussion

Our results demonstrate that oviparous female organisms may exhibit small-scale microhabitat shifts within an oviposition site when laying their eggs. Furthermore, results from a field experiment indicate that these microhabitat shifts render their eggs less vulnerable to predation and thereby enhance the survival of their offspring. Caddisflies are major predators of the egg stage of *T. granulosa*. Over three-quarters of a million caddisflies may occupy a single breeding pond, and, under optimal conditions, could consume the entire reproductive output of the newt population in a pond in as little as 36 h (Gall et al. 2011). This propensity to consume newt eggs is compounded by caddisflies attraction to chemical cues emanating from gravid female newts and eggs, which likely further exacerbate the predation pressure exerted on the newt population. Although caddisflies were attracted to chemical cues from gravid female newts and newt eggs, the chemical involved remains unknown. Tetrodotoxin is an important olfactory cue in some organisms (Matsumura 1995; Zimmer et al. 2006), but was not attractive to caddisflies in this study. The role of TTX in olfaction is likely highly species-specific, as recent work on snakes (*Thamnophis sirtalis*) also failed to find evidence for a role of TTX in chemoreception (Avila et al. 2011).

Non-genetic maternal effects, such as micro-oviposition avoidance, can have dramatic effects on offspring fitness. In predator–prey systems, selection on early life-history stages is intense (Orians and Janzen 1974), and any adjustment in the location of eggs that results in greater offspring survival is likely to be adaptive. For oviparous organisms with access to discrete habitat patches that vary in predation risk, shifting oviposition to habitats lacking egg or larval predators can maximize offspring survival. For example, mosquitos avoid laying eggs in pools with predators (Chesson 1984; Petranka and Fakhoury 1991), and many amphibians have been documented to utilize similar behavior (Resetarits and Wilbur 1989; Crump 1991; Resetarits 1996; Orizaola and Brana 2003). Many oviparous organisms, however, cannot move to a predator-free habitat to lay their eggs. Oviposition sites are often limited (Village 1983; Lancaster et al. 2010), and movement between discrete locations may not be possible due to energy constraints or time limitations (Cappuccino and Kareiva 1985; Rosenheim et al. 2008). Furthermore, moving to a new egg-laying site may invoke risk of mortality for a female from predation or environmental stress (Scheirs et al. 2000; Spencer 2002; Refsnider and Janzen 2010). Although patterns of predator avoidance are clear when choosing between discrete sites, this process, whereby organisms move into enemy-free space to reduce predation risk (Jeffries and Lawton 1984), may be equally common in cases that occur at much smaller spatial scales. Environments are heterogeneous (Pianka 1966; Ricklefs 1977) and, given the importance of offspring survival, any fine-scale adjustment in oviposition choice that fails to increase female fitness relative to other phenotypes is likely to be selected against (as long as there is a genetic basis for the behavior). In our system, caddisflies are highly mobile as adults (winged) and are ubiquitous in most freshwater ecosystems (Wiggins and Currie 2008). It is therefore unlikely that female newts would be able to find a new pond that lacked these predators. However, larval caddisfly locomotion is generally limited due to the presence of a portable case (Dodds and Hisaw 1925), and many species are restricted to benthic habitats (Mackay and Wiggins 1979). In this study, caddisfly abundance decreased with increasing plant height, indicating that *L. flavastellus* does not commonly use the upper portions of aquatic vegetation. The absence of caddisflies in the upper portion of the water column creates an optimal microhabitat for newt oviposition. In this case, spatial variation in predation pressure has probably facilitated the evolution of behavioral responses to avoid egg predators and increase female fitness without the need to find a completely new oviposition site.

The role of enemy-free space in facilitating a shift in oviposition behavior has been well documented in some organisms. Many lepidopteran butterflies shift between host plants in response to predation pressure on eggs or larvae (Singer et al. 2004; Wiklund and Friberg 2008). Murphy (2004) measured the survival and growth of Alaskan swallowtail butterfly larvae on three host plants and found support for the role of enemy-free space in maintaining a host shift in this species. Nevertheless, within-host shifts in oviposition location (i.e. micro-oviposition avoidance) in insects are equally probable when variation in reproductive success exists at a small scale. For example, Lucas and Brodeur (1999) demonstrated that ovipositing female midges (*A. aphidimyza*) do not distinguish between potato plants with or without predatory coccinellids. Females do, however, differentiate between individual leaves on the host plant that have different trichome densities, and ultimately lay more eggs where trichome density is higher; leaves with more trichomes provided greater protection to the embryos in experimental dishes (Lucas and Brodeur 1999). Some butterfly species deposit their eggs on the substrate surrounding their host plant, rather than on the host itself (Wiklund 1984; de-Silva et al. 2011), and experimental evidence indicates that these off-host eggs are more likely to survive (de-Silva et al. 2011). These results provide support that micro-oviposition avoidance can result in elevated offspring survival without the evolution of host-switching.

Although micro-oviposition behavior may be most obvious in predator–prey systems, this process is likely to be adaptive in other contexts, including in response to parasitism or where food quality or abiotic characteristics are variable across microhabitats. For example, female water striders adjust the depth of their eggs in response to a parasitic wasp, despite a trade-off with mortality due to increased water pressure (Hirayama and Kasuya 2009, 2010). Birds and reptiles are well documented to exhibit nest-site preferences that are dependent on microclimatic variables (Shine and Harlow 1996; Wilson 1998; Lloyd and Martin 2004), and some female butterflies select plant parts that optimize thermal conditions (e.g. Williams 1981; Grossmueller and Lederhouse 1985). Although newts responded to the presence of caddisflies by depositing more eggs in the upper sections of a vertical chamber, a general upward bias was observed and may be due to several factors. An upward bias during egg laying has been observed in other newt species and may be due to oxygen requirements of the female (Miaud 1995), or to expose the embryos to warmer temperatures. Alternatively, past selective pressure by caddisflies resulting in reduced survival of eggs on lower vegetation may have resulted in the partial genetic fixation of this behavioral response. Regardless, female newts showed a significant increase in oviposition height in response to the presence of caddisflies, indicating that a general preference to elevate eggs is compounded by a behavioral response to reduce predation.

The behavioral plasticity exhibited by females to reduce predation risk to their offspring is one example of the class of phenotypes that are expected to evolve through maternal selection (Kirkpatrick and Lande 1989; Cheverud and Moore 1994; Wolf et al. 1998; Wolf and Wade 2009). Such traits influence maternal fitness indirectly through their impact on offspring fitness. Unlike maternal effect traits, maternal selection traits do not alter offspring phenotypes, but rather influence offspring fitness directly. The resultant transgenerational effect on maternal fitness is expected to lead to the evolution of a suite of egg-protecting behaviors. Although identifying additional partitioning of female oviposition avoidance behavior into microhabitat variables may be difficult in some cases (and may not occur in others), such behavior may be an important source of variation in female fitness. The comprehensive results on amphibians presented in this study, combined with previous work on insects (Lucas and Brodeur 1999; Hirayama and Kasuya 2009, 2010), suggest that fine-scale selection by ovipositing females (i.e., micro-oviposition avoidance) may be a common feature of the oviposition decisions of many terrestrial and aquatic oviparous organisms.
